# Screening of Marine Bacteria for Lipase Activity and Application as Whole-Cell Biocatalysts

**DOI:** 10.3390/microorganisms14061355

**Published:** 2026-06-17

**Authors:** Luís C. de Sousa, Ana J. Caeiro, Carla C. C. R. de Carvalho

**Affiliations:** 1 Department of Bioengineering, iBB-Institute for Bioengineering and Biosciences, Instituto Superior Técnico, Universidade de Lisboa, Av. Rovisco Pais, 1049-001 Lisbon, Portugal; luis.c.de.sousa@tecnico.ulisboa.pt (L.C.d.S.);; 2 Associate Laboratory i4HB-Institute for Health and Bioeconomy, Instituto Superior Técnico, Universidade de Lisboa, 1049-001 Lisbon, Portugal

**Keywords:** lipase, marine bacteria, bioprospecting, culture-based screening, whole-cell biocatalyst

## Abstract

Several strategies can be employed for the identification of novel microbial lipases. Despite the increasing importance of metagenomics in bioprospecting, significant limitations in the expression of recombinant proteins, and lipases in particular, remain. Culture-based bioprospecting approaches are, therefore, still valuable. In this work, a collection of bacterial isolates, mainly of marine origin, was screened for lipase activity through a culture-based approach. Screening for lipolytic bacteria was performed in solid media containing olive oil emulsions and rhodamine B. Positive isolates were subsequently grown in liquid media, to confirm lipase production. Significant hydrolytic activity towards the triglyceride substrates tributyrin and triolein could be observed with the biomass produced, although no lipase activity could be detected in the culture supernatants. Six isolates presenting high activity were characterized as whole-cell biocatalysts, and all were found to be active at temperatures ranging between 25 and 65 °C, and at pH values between 6 and 10.5. Genomic analyses of two of these Gram-negative lipase-producing isolates revealed the presence of several hypothetical genes encoding for lipolytic enzymes, including outer cell-bound enzymes, predicted through the application of machine-learning tools. These natural isolates, containing cell-associated lipases, may therefore be of special interest for application as whole-cell biocatalysts.

## 1. Introduction

The introduction of novel biocatalysts for the production of consumer products, and as part of their formulation, are two of the many solutions required for a more sustainable future [1]. Indeed, despite the application of biocatalysis in artificial processes predating the definition of concepts like catalysis, protein, or enzyme by millennia [2,3], new applications of enzymes are still being described. Lipases, or triacylglycerol acylhydrolases (EC 3.1.1.3) [4], the focus of this work, have been applied to a wide range of industrial processes [5,6,7]. These applications rely on the wide variety of reactions potentially catalysed by these enzymes. Lipases are generally defined by catalysing the hydrolysis of medium- and long-chained, water-insoluble, acylglycerols [8,9]. The addition of lipases in detergent products, first suggested in the early 20th century [10,11], but only achieving generalized application by the 1990s [12], is based on these hydrolysis reactions. Additionally, lipases are often able to catalyse a wide variety of esterification and transesterification reactions when used in organic solvent systems [13,14,15,16], often presenting stereoselectivity in these reactions [17]. These characteristics allow for the application of lipases in different organic synthesis reactions [18], such as the production of structured lipids [19], or of optically pure active compounds for drug production [20].

Lipases generally present significant overlap in substrate selectivity with other hydrolases of carboxylic esters, making the classification of these enzymes somewhat troublesome. Esterases, for example, commonly share substrates with lipases, but are generally limited to the hydrolysis of water-soluble esters [21]. Other common distinctions from esterases, such as the presence of lid domains in (many) lipases [22], or lipases presenting interfacial activation, i.e., increased activity towards micellar, emulsified, or otherwise saturated substrates versus monomeric substrates [8], are important characteristics in many lipases, but not universal in this class of enzymes [23]. Other hydrolases of carboxylic esters, e.g., cutinases [24] or phospholipases [25], may hydrolyse water-insoluble acylglycerols, although activity towards these substrates will not be present in all members of these enzyme classes [25]. Nevertheless, this overlap in activities may lead to the designation of an enzyme as a lipase, without this classification presenting significant relevance to the biological role of the enzyme in its native host, if a limited selection of “conventional” lipase substrates is used for the characterization of the hydrolase. Regardless, this ambiguity in classification does not limit the use of these enzymes in industrial applications.

Several strategies have been employed in the bioprospecting of microbial lipases, based on culture-independent metagenomic methodologies [26,27,28], or on culture-dependent methodologies [29,30,31,32,33]. Metagenomics have increasingly become important in enzyme bioprospecting [34] due to the improved availability of next-generation sequencing technologies, the development of new bioinformatic tools [35,36], and high-throughput screening strategies [37]. Metagenomic strategies can offer several advantages over culture-based strategies [26,38]. For instance, they allow for the identification of biocatalysts in yet-to-be-cultured organisms, although the magnitude of this advantage is often overstated due to the severe underestimation of the number of organisms that can be grown under laboratory conditions [39]. Additionally, the availability of an increasing number of metagenomes in public repositories opens the number of resources available to be screened, generating an increasing gap between hypothetical enzymes and those with validated activity [38]. However, the expression of recombinant enzymes in traditional hosts such as *Escherichia coli* often leads to inactive proteins, a phenomenon designated as the “great expression inability” by Ekkers et al. [40]. Indeed, the expression of bacterial lipases often exemplifies this issue, with some of these enzymes, particularly those belonging to lipase families I.1 and I.2, requiring specific chaperones to achieve a final, active conformation [9,23], although previous functional metagenomics studies have been able to express chaperone-dependent lipases by simultaneously expressing both genes [41,42]. Nevertheless, expressing lipases in their native hosts aids in ensuring the production of active enzymes, and thus culture-based bioprospecting can still be a valuable strategy in the omics era.

Marine environments have proven to be valuable sources for novel biocatalysts and other bioactive compounds [43,44,45]. The selective pressures imposed on microorganisms by these environments, including high salinity, high pressure, and extreme temperature (both low temperatures and high temperatures associated with geothermal activity), often translate to wide tolerance to these factors by the enzymes/biocatalysts produced by those organisms [45]. Several microbial lipases, both of bacterial and fungal origin, have been identified previously from marine environments [46,47], showing important roles in the trophic chains of these environments [48,49].

Over the last decade, our group has organized a collection of over a thousand microbial isolates, mostly from marine environments (but also including other aquatic sources). The sampling campaigns used in the collation of this collection have allowed, for example, for the identification of a prodigiosin-producing *Serratia rubidea* [50], a melanin-producing yeast [51], trimethylamine-degrading bacteria [52], and several other biocatalysts [53,54], by culture-based approaches. We hypothesized that the bacterial isolates from marine habitats we sampled would exhibit lipolytic activity compatible with industrial requirements, and therefore designed this work with the objective to screen part of the collection to find the most suitable whole-cell biocatalysts for further biotechnological applications. Indeed, several isolates were identified as lipase producers. Of these, two were selected to have their whole genome sequenced, to identify the putative lipases responsible for the activity of these microorganisms.

## 2. Materials and Methods

### 2.1. Materials and Reagents

Marine agar (MA) and marine broth (MB) were purchased from Condalab (Madrid, Spain). Gum arabic from acacia tree, olive oil (highly refined, low acidity), *p*-nitrophenyl butyrate (*p*NPB), 2-morpholinoethanesulfonic acid monohydrate (MES), 1,4-piperazinediethanesulfonic acid (PIPES), 3-(cyclohexylamino)-1-propanesulfonic acid (CAPS), sodium chloride, sea salts, ferric citrate, and phenol red were all obtained from Sigma-Aldrich (St. Louis, MO, USA). Rhodamine B and tributyrin were purchased from Carl Roth (Karlsruhe, Germany). Trypticase soya broth (TSB), granulated agar, and yeast extract were acquired from BD (Sparks, MD, USA). Glucose was purchased from Scharlau (Sentmenat, Spain). Meat peptone and butyric acid were obtained from ApplieChem Panreac (Darmstadt, Germany). 4-(2-Hydroxyethyl)-1-piperazinepropanesulfonic acid (EPPS) was purchased from Acros Organics (Geel, Belgium). Trioctanoin and octanoic acid were obtained from TCI Chemicals (Tokyo, Japan), and 2-(cyclohexylamino)ethanesulfonic acid (CHES) was acquired from Thermo Scientific (Ward Hill, MA, USA). Tris(hydroxymethyl)aminomethane (Tris) was purchased from Eurobio Scientific (Courtaboeuf, France). Dimethyl sulfoxide (DMSO) and acetonitrile were purchased from Fisher Scientific (Loughborough, UK). 

### 2.2. Isolate Selection and Cultivation

The production of lipases was assessed by screening 94 bacterial isolates. Isolates originated from our collection, having been recovered from samples collected at multiple locations (Table 1). Isolates are identified throughout this work by a number, corresponding to the order of their addition to the collection. Isolates were stored at −80 °C, in TSB or MB containing 20% (v/v) glycerol, and recovered by inoculation on MA or on TSB containing 1.5 g L^−1^ agar (trypticase soy agar, TSA), and subsequently incubated at 30 °C.

### 2.3. Screening of Isolates for Lipase Production

Isolates were screened for lipase production by the presence of fluorescence in MA plates supplemented with 2% (v_emulsion_/v_media_) of an emulsion of olive oil with gum arabic, containing rhodamine B, in a procedure adapted from Kouker and Jaeger [55] and Molitor et al. [56]. The olive oil emulsion was prepared by adding 50% (v/v) of olive oil to a solution containing 100 g L^−1^ gum arabic and 0.7 g L^−1^ rhodamine B, prepared in sterile de-ionized water, followed by vigorous mixing with a vortex mixer. The emulsion was then added to sterile, molten MA, at a ratio of 2% (v_emulsion_/v_media_). The media was mixed thoroughly with a magnetic stirrer while cooling, before being distributed in four-compartment quadriPERM plates (Sarstedt, Nümbrecht, Germany). The resulting media was pink in colour, due to the addition of rhodamine B, and translucent, due to a combination of the emulsion and the regular turbidity of the MA medium. Screening plates were streaked with isolates grown overnight in either MA or TSA, one isolate per quadriPERM plate compartment. Plates were then incubated at 30 °C for up to a week. Plates were photographed daily under visible and ultraviolet (UV) light using a GenoSmart imaging system (VWR, Radnor, PA, USA). Any isolate presenting fluorescence under UV light, in or around the bacterial grown cells, at any point during the incubation, was considered to be a presumptive lipase producer.

### 2.4. Validation of Lipase Production

Lipase production in isolates identified as positives in the previous screening was validated by their cultivation in liquid media, and subsequent measurement of the lipase activity of both the culture supernatant and biomass. Four liquid culture media were tested in these assays: MB; TSB; TSB + 14.4 g L^−1^ NaCl; and a novel medium composition, hereafter designated as SPGY. The latter contained sea salts at 40 g L^−1^, meat peptone at 5 g L^−1^, glucose at 5 g L^−1^, yeast extract at 1 g L^−1^, ferric citrate at 0.1 g L^−1^, olive oil at 0.5% (v/v), 1.55 g L^−1^ K_2_HPO_4_, and 0.85 g L^−^^1^ NaH_2_PO_4_. One or more media compositions were tested for each isolate, depending on their ability to grow in the different media. Liquid cultures were carried out in 100 mL shake flasks, containing 20 mL of cultivation media. Shake flasks were inoculated at 10 % (v/v), with inocula grown overnight in the same media, and under the same conditions as the main cultures. Cultures were incubated in orbital shakers (Argitorb 200 from Aralab, Rio de Mouro, Portugal) at 30 °C and 200 rpm, for up to 24 h. After harvesting, culture biomass and supernatants were separated by centrifugation on a 5810 R centrifuge (Eppendorf, Hamburg, Germany) at 2800× *g*, for up to 30 min, until a clear supernatant was obtained. Supernatant was then recovered, and the remaining biomass pellet was washed twice with a 0.15 M NaCl solution. After discarding the washing supernatant, the biomass pellet was suspended in buffer to an optical density at 600 nm of 40, for application in lipase activity assays.

Lipase activity assays were performed with the pH indicator-based lipase assay (PHIBLA) method, using tributyrin and trioctanoin as substrates [57,58]. Phenol red and EPPS buffer were employed as the pH indicator/buffer pair [59]. In 96-well microplates, the following solutions were sequentially added: (i) 100 µL of a buffer and substrate mixture, containing 90.9 µL of EPPS buffer at 2.5 mM, pH 8, and 9.09 µL substrate, tributyrin or trioctanoin, at 100 mM in DMSO; (ii) 90.9 µL of 0.304 mM phenol red in EPPS buffer at 2.5 mM, pH 8; (iii) and 9.09 µL of biomass suspension or culture supernatant, to start the reaction. Solution (i) was vigorously mixed with a vortex mixer for 15 s before being used. Microwell plates were incubated at 30 °C for up to 90 min. Every 2 min, the absorbance of the solution at 550 nm was measured, after shaking for 10 s. Incubation and absorbance measurements were performed on a Multiskan GO microplate reader (Thermo Scientific). Control reactions, replacing solution (iii) with buffer, were performed simultaneously, and the resulting absorbances at 550 nm were subtracted from the results of the assays. Reactions were performed in triplicate. Activity was determined by the slope of the absorbance at 550 nm plotted over time, after conversion to butyric/octanoic acid concentration through external calibration curves. The calibration curves were created in media with the same composition as the control reactions, but replacing the substrate with solutions of butyric acid (dissolved in water) or octanoic acid (dissolved in DMSO), to achieve concentrations in the reaction media ranging between 1.357 mM and 0.027 mM, for which absorbance at 550 nm presented a linear relation with free fatty acid concentration. Calibration curves were repeated for every buffer/indicator solution, to accommodate for small variation in concentrations between preparations. One enzyme activity unit (U) was defined as the amount of enzyme catalysing the production of 1 µmol of butyric/octanoic acid per minute.

### 2.5. Characterization of Whole-Cell Biocatalysts

Optimal pH and temperature for whole-cell biocatalysis were determined for isolates 14, 701, 705, 725, 729, and 790, with *p*NPB as substrate [60]. Reactions were performed in 96-well microplates, with 200 μL of reaction media, composed of: 180 μL of buffer; 10 μL of biomass suspension, prepared as described previously; and, 10 μL of *p*NPB at 20 mM in acetonitrile. For the determination of temperature optima reactions were performed in a range of 25 °C to 65 °C, at pH 8, in Tris-HCl buffer at 50 mM. For the determination of pH optima, reactions were performed at 30 °C, with the following series of buffers at 50 mM: MES buffer, pH 6 and 6.5; PIPES buffer, pH 6.5, 7, and 7.5; Tris-HCl buffer, pH 7.5, 8, and 8.5; CHES buffer, pH 8.5, 9, and 9.5; and CAPS buffer, pH 9.7, 10, and 10.5. Reactions were started by the addition of substrate, and subsequently monitored for 20 min by measurements of absorbance at 348 nm (isosbestic point of *p*-nitrophenol/*p*-nitrophenolate, ε = 5.617 mM^−1^ cm^−1^) performed every 30 s by a Victor Nivo microplate reader (PerkinElmer, Shelton, CT, USA). One enzyme activity unit was defined as the amount of enzyme catalysing the production of 1 µmol of *p*-nitrophenol/*p*-nitrophenolate per minute, as determined by the rate of production of the compounds, after subtraction of their rate of production in control reactions without enzyme. Reactions were carried out in triplicate.

### 2.6. Taxonomic and Genomic Analysis of Isolates

#### 2.6.1. Sherlock^®^ Microbial ID System

Isolates, screened as potential lipase producers in MA supplemented with an olive oil emulsion, were identified based on their fatty acid profiles with the Sherlock^®^ Microbial ID System from MIDI, Inc. (Newark, DE, USA). For this, isolates were streaked in TSA and incubated at 30 °C for 24 h ± 1 h; isolates that showed no growth in TSA were excluded from the analysis. Lipids from the resulting biomass were extracted and derivatized into fatty acid methyl esters (FAMEs) by the Instant-FAME^TM^ procedure, according to manufacturer instructions [61]. FAMEs were then analysed by gas chromatography on an Agilent 6890N gas chromatograph with flame ionization detector (GC–FID), equipped with an 7683B Series autosampler, and a J&W Ultra 2 phenyl methyl siloxane capillary column from Agilent (Santa Clara, CA, USA), with a column length of 25 m, inner diameter of 200 µm, and film thickness of 0.33 µm. GC–FID analysis of FAMEs, and subsequent identification of isolates, were performed with the ITSA1 GC analysis method and associated identification library for environmental isolates [61], with the Sherlock^®^ software suite, version 6.2, from MIDI, Inc. Principal component analysis (PCA) of the fatty acid profiles of the isolates was also performed with the Sherlock^®^ software, by application of the PCA model generated for the fatty acid profiles in the identification library (PCA model is included in the software).

#### 2.6.2. Sequencing of 16S rDNA

Isolates 14, 701, 705, 725, 729, and 790 were also identified based on the sequence of their respective 16S rRNA genes. DNA extraction was performed with the Qiagen DNeasy UltraClean Microbial Kit (Qiagen, Hilden, Germany), according to manufacturer instructions. Amplification, and subsequent Sanger sequencing, of 16S rDNA fragments between variable regions V1 and V9 was performed by STAB VIDA (Almada, Portugal). Isolates were subsequently identified based on BLASTN version 2.17.0+ (NCBI, Bethesda, MD, USA) searches of the sequences.

#### 2.6.3. Whole-Genome Sequencing

Isolates 14 and 790 were further characterized by whole-genome sequencing. Genomic DNA was extracted with the Qiagen DNeasy UltraClean Microbial Kit. Genome sequencing and assembly were performed by STAB VIDA: sequencing was performed with an Illumina NextSeq platform, with 300 base pairs (bp), paired-end reads; read trimming and de novo assembly were performed with CLC Genomics Workbench, version 12.0.3, from Qiagen. Quality assessment of the assemblies was performed with QUAST, version 5.2.0 [62]. Genome annotation was performed with the Bakta Web platform [63,64]. Predicted products of coding sequences annotated as lipases, esterases, α/β-hydrolases, and GDSL/SGNH hydrolases, were further classified with the HMMER tool, version 2.3.2 [65], available in the ESTHER database [66] (“HMMER tool”, mentioned throughout this work, only refers to its implementation in the ESTHER database). Select products, lacking identification with the HMMER tool, were alternatively analysed with the InterProScan tool [67], available in the InterPro platform [68]. The subcellular location of hypothetical lipolytic enzymes was predicted with a combination of DeepLocPro, version 1.0 [69], and SignalP, version 6.0 [70]. Amino acid residue sequences of predicted lipases were also searched with BLASTP version 2.17.0+ (NCBI), against the non-redundant protein sequences database.

## 3. Results and Discussion

### 3.1. Screening of Isolates for Lipase Production

**Figure 1:**
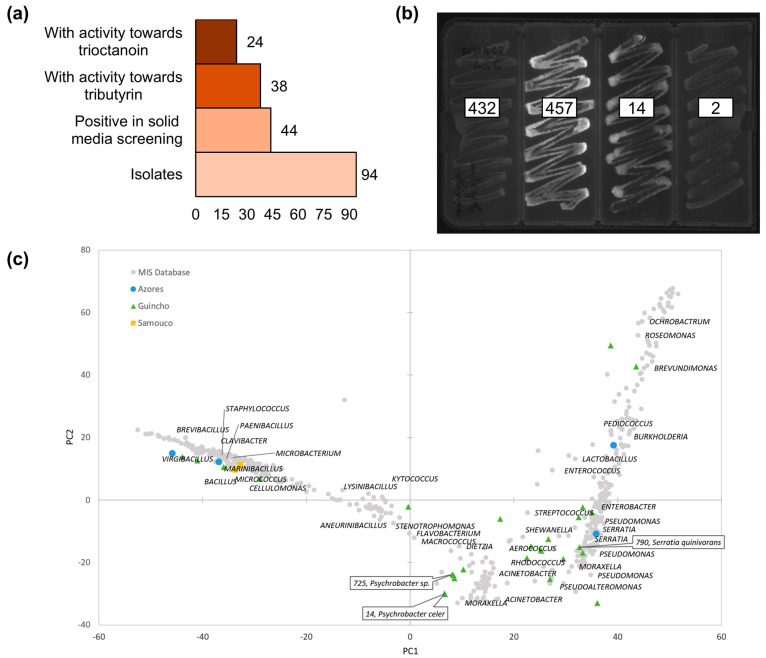
Screening of isolates for the production of lipases. (**a**) Compilation of results of screening assays: (i) screening in solid media plates containing an olive oil emulsion, and rhodamine B as indicator for acylglycerol hydrolysis; (ii) screening of isolates for esterase/lipase activity in biomass, with tributyrin/trioctanoin as substrates, using a threshold of specific activity of 1 mU mg_biomass_^−1^ for identification as a positive. (**b**) Example of solid media screening plate, photographed under UV light after 96 h incubation at 30 °C, with isolates 457 and 14 being identified as positive for lipase production. (**c**) PCA analysis of fatty acid profiles of isolates identified as positive for lipase production in solid screening media assays, highlighting representative genera, and positive isolates subsequently identified by sequencing of 16S rDNA; analysis of fatty acids and PCA were performed using the Sherlock^®^ Microbial ID System, with the ITSA1 method/database.

In this work, 94 isolates were screened for the production of lipases. Although the majority of these isolates originated from temporary rock pools from the same coastal environment, several isolates from other sources were also screened, representing the diversity of sources of the isolates present in our collection. Initial screening for lipase production was performed in marine agar plates containing emulsions of olive oil and rhodamine B (Figure 1a,b). Rhodamine B becomes fluorescent under UV light in the presence of free fatty acids [55], therefore allowing for the detection of the hydrolysis of the emulsified olive oil, and thus the assessment of the production of lipases by the isolates inoculated in the media. During the screening, 44 isolates presented fluorescence under UV light, in a period of up to a week ofincubation. A PCA analysis of the fatty acid profiles of the positive isolates was conducted to help in elucidating their identity (Figure 1c) [39,71]. Isolates unable to grow in TSA (701, 705, 708, 709, 727, 728, 729, 739, 747, 752, 754, and 793) were excluded from the analysis, as growth in this medium is required for identification with the Sherlock^®^ Microbial ID System, when applying the ITSA1 method/database. Lipase producers were clustered with multiple phyla, in accordance with the wide distribution of these enzymes, not only in Bacteria, but in all domains of life [9,72,73]. For instance, isolates 232, 562, 771, 774, 776, 795, 901, and 903 were found to present great proximity with representative genera of Gram-positive phyla *Bacillota* and *Actinomycetota*, with both phyla containing lipase-producing species [9,74,75,76,77]. Other isolates showed proximity with different clades, in particular with *Enterobacteriaceae*, and other families of phylum *Pseudomonadota*, also well-represented in previous works discussing bacterial lipases [9,75,76,78].

**Figure 2:**
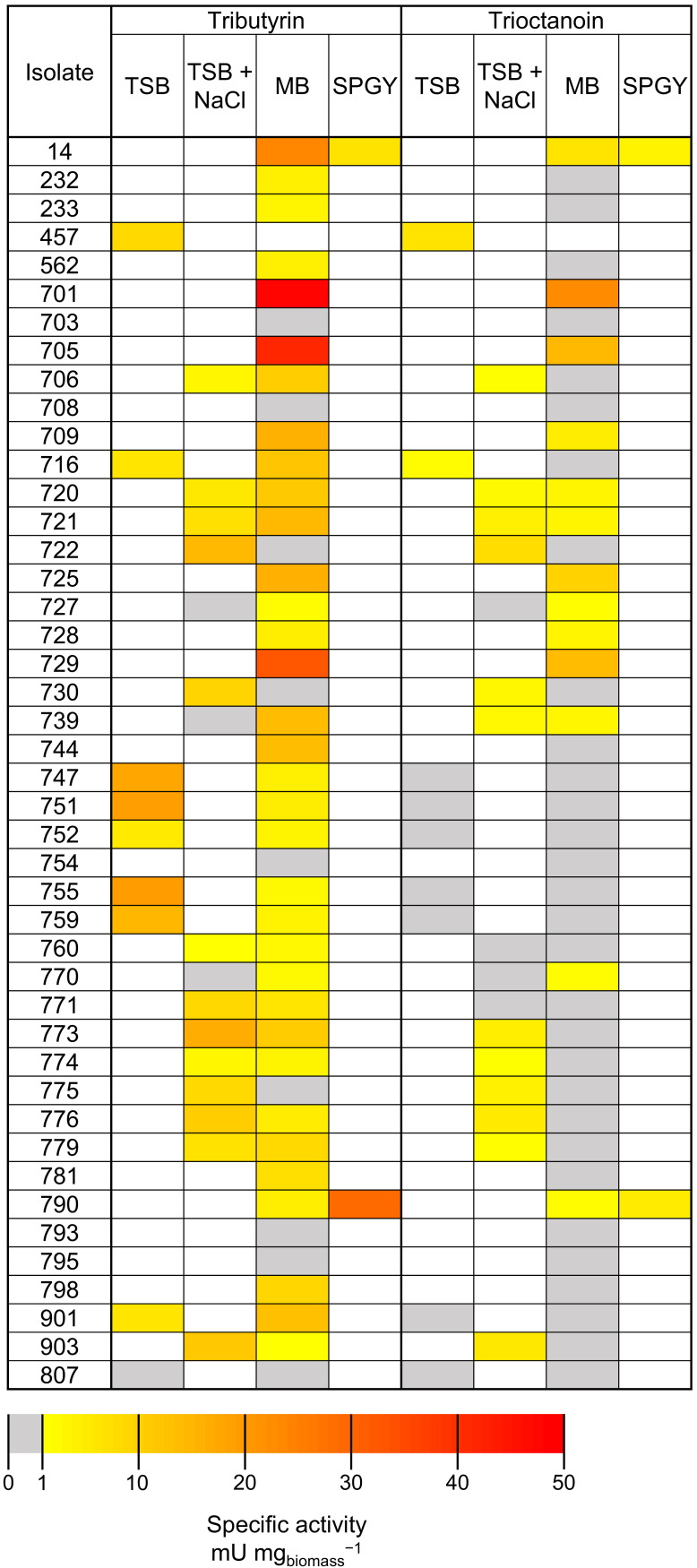
Heatmap of specific activities in biomass (mU mg_biomass_^−1^), from liquid cultures of 44 isolates, identified as lipase producers in solid media screenings, with tributyrin or trioctanoin as substrates. Isolates were grown in one or more of the following media: TSB, TSB supplemented with 14.4 g L^−1^ of salt (TSB + NaCl), MB, or SPGY. White spaces identify media compositions, for a given isolate, for which lipase activity was not determined. Activities lower than a threshold of 1 mU mg_biomass_^−1^ were considered to be null and are marked in grey. Values represented correspond to the average of three replicates.

Isolates identified in solid media screens as positive for lipase production were subsequently grown in liquid media, to confirm the production of the enzymes. Supernatant and biomass resulting from these cultures were recovered and tested for the presence of (i) free extracellular lipases and (ii) cell-bound lipases, respectively. Lipase activity assays were performed according to the PHIBLA method, with tributyrin and trioctanoin as substrates [58]. This substrate selection allows for the discrimination between the production of lipases and esterases: the partially water-soluble tributyrin can frequently be hydrolysed by both classes of enzymes, while trioctanoin, with its minimal water solubility, can generally only be hydrolysed by lipases [21]. Significant activity, both with tributyrin and trioctanoin as substrates, was detected on the biomass of the cultures of most of the positive isolates (Figure 1a and Figure 2). However, no activity could be detected in the corresponding culture supernatants, either due to the lack of expression of these proteins, or due to their inactivation.

Cell-bound hydrolases play a crucial role in the carbon metabolism of marine bacteria, as the few carbon sources that are available in these environments are often polymeric substances that must by hydrolysed to allow for their uptake by the cells [79]. Retaining enzymes in close proximity to the cell may be especially important to pelagic (and other planktonic) bacteria, as they lack the barriers to diffusion of enzyme and hydrolysis products afforded by the production of biofilms [80,81]. These factors may therefore partially explain the lack of free lipase activity, as they may favour the repression of free lipase expression as an adaptation to growth in liquid culture. In accordance to this, cell-bound lipase activity has been observed to be common in marine bacteria [82].

Culture media had a significant effect on the expression of cell-bound lipases (Figure 2). Similar effects have been observed previously, with lipase production by bacteria varying greatly with culture composition, e.g., carbon and nitrogen sources [83,84,85,86], but also with incubation parameters such as temperature [87]. It has been described previously that while TAG can act as inducers of lipase production in bacteria [88], this effect is not universal [83,89]. For instance, the expression of extracellular lipase LipA in *Acinetobacter calcoaceticus* was found to be very reduced when triolein was used as the sole carbon source, when compared with hexadecane [90]. This effect was partially linked to the observation that oleic acid, resulting from the hydrolysis of triolein by the growing culture, inhibited the expression of the lipase [90]. Similar inhibitions of lipase expression by long-chained free fatty acids have been described previously [89,91], while the reverse effect has also been observed, for example in *Pseudomonas aeruginosa* strain YS-7 [88]. Lipolytic enzymes in bacteria perform multiple physiological functions beyond carbon source metabolism, including acting as virulence factors in animal [92,93,94,95] and plant pathogenicity [96]. In accordance to this, lipase expression in bacteria is often regulated by multiple mechanisms, including by quorum sensing systems [97,98]. Therefore, TAG hydrolysis for carbon and energy metabolism should not be taken as the only possible physiological role of a bacterial lipase, and hence TAG supplementation as a necessary factor in expressing these enzymes. Nevertheless, it remains a possibility that supplementation of culture media with TAG, or other potential lipase substrates such as polysorbates (which are fatty acid ester-based surfactants/emulsifiers commercialized as Tween), which are often found to induce lipase production in bacteria [88,89,99], could have led, in some isolates, to the production of free extracellular lipases, or increased production of cell-bound lipases. The presence of six isolates, screened as positive for lipase production in solid media, that in liquid cultures did not produce lipases, and the 14 isolates that only expressed esterases in liquid culture, may therefore have been the product of a lack of appropriate inducers in the media. It should be noted, however, that experiments with supplementation of culture media with the lipase substrate olive oil led to no improvement in the activity of isolate 14, or to the presence of free lipase activity, although it led to an increase in cell-bound activity of isolate 790 towards tributyrin, with a small effect on activity with trioctanoin.

In this study, no determination could be made in terms of substrate selectivity between tributyrin and trioctanoin, based on the sole activities measured for the two substrates. Lipases are interfacial enzymes, active at the interfaces of water-insoluble substrates, meaning that the available area of interface present in the reaction media, and the characteristics of that interface, play a major role in the availability of the substrate, and therefore in the activity measured in an enzymatic assay [100]. Given the different characteristics of the two substrates [101], particularly their solubility/miscibility in water, distinct emulsions, with different droplet distributions, were likely formed for tributyrin and trioctanoin. These factors preclude any determination of the preferential substrates for these enzymes (e.g., short- versus medium-chain TAG), based on the activity measurements obtained with the PHIBLA method as applied in this work, in the reactions using the two substrates.

Despite the lack of enzyme activity on culture supernatants, the lipolytic activity observed on culture biomass should still be the result of extracellular proteins, that alternatively remained associated with the enzyme-producing cells. Several examples of these proteins have been described previously [102,103,104,105,106]. The presence of cell-bound lipolytic enzymes may potentially allow for the of application of the isolates as whole-cell biocatalysts, if other characteristics such as stability under reaction conditions, and activity towards relevant substrates, are also observed. Generically, the application of whole-cell biocatalysts can offer several advantages over purified enzymes, including reduced production costs [107,108,109], thus increasing their industrial desirability. One often-suggested application of whole-cell biocatalysts is the production of biodiesel, by (trans)esterification reaction, with organic solvent-tolerant organisms expressing lipases [110,111,112]. Whole cell-biocatalysis processes, relying on organisms that naturally express lipases, have been described previously [106,113,114]. Recombinant protein technology has also been employed for the production of membrane-bound lipases, for instance by the construction of fusion mutants of lipases with autotransporter domains [115], or with other membrane protein domains [116]. For example, co-expression of a lipase and its corresponding chaperone, from *Burkholderia cepacia*, as mutant proteins with fused with autotransporter domains, in *E. coli*, allowed for the production of active membrane-bound lipases. The resulting whole-cell biocatalysts were shown to perform well in standard laundry-washing tests, with oil-soiled textiles [117].

### 3.2. Characterization of Lipolytic Whole-Cell Biocatalysts

Six isolates were selected for further characterization, since they presented the highest specific activities with tributyrin and/or trioctanoin, namely, isolates 14, 701, 705, 725, 729, and 790 (Table 2). These isolates were identified by sequencing of 16S rDNA. All isolates were Gram-negative *Gammaproteobacteria*, recovered from two distinct sampling campaigns of the same temporary rock pools from a coastal environment (Guincho-Cabo Raso, Portugal). Three *Pseudoalteromonas* isolates, 701, 705, and 729, had not been identified previously by the Sherlock^®^ Microbial ID System, as they did not grow in TSA and could not be compared to the database. The three *Pseudoalteromonas* isolates presented different colony phenotypes when grown in marine agar: isolate 701 presented large pink colonies, isolate 705 formed small yellow colonies, whilst isolate 729 formed small white colonies. Phenotype variability in *Pseudoalteromonas* colony colour is a hallmark of this genus. Additionally, isolates 705 and 729 were found to share 98.86% similarity between the 16s rDNA sequences, which together with colony phenotype, allows for their classification as distinct but co-occurring strains of *P. undina*. Isolate 725, identified as *Psychrobacter* sp., presented high proximity to isolates 720 and 721 in the PCA analysis of fatty acid profiles (Figure 1c), and similar activities with tributyrin (Figure 2), indicating that these are likely closely related strains. Isolate 14, *Psychrobacter celer*, also showed close proximity to these isolates. Isolate 790, *Serratia quinivorans*, presented proximity to isolate 781, which only produced esterases in the liquid culture assays.

Isolates 14, 701, and 705 presented similar specific growth rates in MB, which were higher than those of the remaining isolates (Table 2). Nevertheless, isolate 790 presented the highest biomass concentration in liquid culture, and subsequently the highest volumetric activity with tributyrin as substrate. However, the higher lipase specific activity attained in the *Pseudoalteromonas* isolates, measured with trioctanoin as substrate, compensated for their lower biomass production, leading to higher lipase production titers (i.e., to increased lipase activity per volume of culture, determined with trioctanoin as substrate) than with isolate 790.

**Figure 3:**
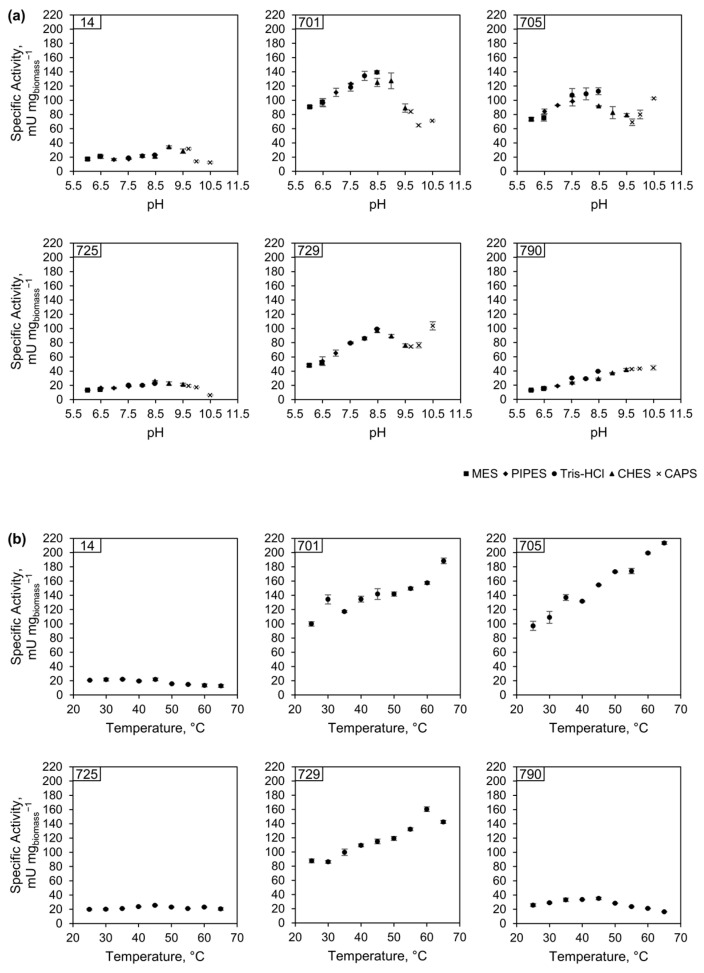
Characterization of whole-cell biocatalysts, with *p*NPB as substrate: (**a**) specific activity in a pH range between pH 6.5 and 10.5, performed with a buffer series of MES, PIPES, Tris-HCl, CHES, and CAPS; (**b**) specific activity in a temperature range between 25 and 65 °C. The isolate number is presented on the top left corner of each graph. Values represent the average of three reactions, after subtraction of the rate of hydrolysis of control reactions performed without enzyme. Data values are represented as mean ± standard deviation.

The range of pH and temperature values where the six isolates, as lipolytic whole-cell biocatalysts, remained active, were subsequently determined with *p*NPB as substrate (Figure 3). It should be noted that this characterization of the whole-cell biocatalysts is distinct from a characterization of purified enzymes, as the presence of the enzymes in a cellular environment may modify their temperatures/pH activity profiles. Additionally, as subsequently discussed, multiple lipases/esterases may be simultaneously expressed by the isolates, and therefore the results of the characterization of the whole-cell biocatalysts may be a combination of characteristics of different enzymes. Whole-cell biocatalysts were tested in temperatures ranging between 25 and 65 °C, and at pH values between 6 and 10.5. Reactions performed at pH 11 presented high rates of non-enzymatic hydrolysis of *p*NPB, precluding accurate measurements of enzyme activity at higher pH. The hydrolysis of *p*NPB was monitored spectrophotometrically at 348 nm, the isosbestic point for *p*-nitrophenol and its conjugate base, *p*-nitrophenolate, thus allowing for the measurement of the formation of the chromogenic compound regardless of the pH of the reaction [118].

Isolates 14 and 725 presented similar activity profiles within the tested pH range (Figure 3a), with a gradual increase in activity being observed up to pH 8.5 for isolate 725, and pH 9 for isolate 14. At pH 6, isolates 14 and 725 retained 50.4% and 51.1%, respectively, of their maximum activity, while at pH 10.5, isolate 14 presented 36.4% and isolate 725 presented 23.9% of their maximum activities. However, the temperature profile for the two biocatalysts differed more significantly. While both isolates presented maximal activity at 45 °C (Figure 3b), isolate 725 presented a larger proportion of residual activity at higher temperatures, retaining 80.3% of its maximal activity at 65 °C, compared to 57.7% observed with isolate 14. Purified lipases from other strains of *Psychrobacter* are often reported to be mildly alkaline [119], with optimal pH values near pH 8 [120,121,122,123], and presenting optimal temperatures of 30 °C [120,121] to 40 °C [122,123].

*S. quinivorans* isolate 790 presented maximum hydrolysis of *p*NPB at 45 °C (Figure 3b). Residual activity (relative to activity at 45 °C) ranged between 73.4% at 25 °C and 46.8% at 65 °C. Hydrolytic activity generally increased with pH, from a residual activity (relative to activity at optimal pH) of 29.1% at pH 6, to a plateau at pH 10.5 (Figure 3a). Several lipolytic enzymes, produced by *Serratia* strains, have been described previously, although the majority of these originate from *S. marcescens*. Several of these lipases from *S. marcescens* have been reported to present optimal activity at 45–50 °C, and at pH 8 [124,125,126]. The same optimal pH was found for a lipase, with polyurethanase activity, isolated from *S. liquefaciens*, although this enzyme had a lower optimal temperature of 30 °C [127]. The optimum temperature for hydrolysis of *p*NPB by isolate 790 is in accordance with what was reported for purified lipolytic enzymes from *Serratia*, but its higher activity in alkaline reaction media appears to be atypical.

The three isolates from genus *Pseudoalteromonas* presented similar temperature and pH profiles (Figure 3), especially isolates 705 and 729. All three isolates presented activity at high temperatures, with optimum temperatures for lipolysis by isolates 701 and 705 likely being higher than 65 °C, outside the range of temperatures tested in this study. On the other hand, isolate 729 presented its maximum activity at 60 °C. Under milder temperatures, the three isolates presented residual activities (relative to the activity measured in their respective optimum temperature) at 25 °C of 53% for isolate 701, 45.5% for isolate 705, and 54.6% for isolate 729. Isolates 705 and 729 each presented two local maxima of activity between pH 6 and 10.5 (Figure 3a). The first “local maximum” occurred at pH 8.5, with the “second maximum” being observed at the end of the pH range tested. This is indicative of the presence of at least two enzymes catalysing the hydrolysis of *p*NPB, with one being significantly more alkalophilic than the other.

Reaction pH did not produce the same effect on the lipolytic activity of isolate 701: maximum activity was reached at pH 8.5, but a “stabilization” in the decrease of activity at higher pH values occurred between pH 10 and 10.5 (Figure 3a). Previously described esterases and lipases from members of the *Pseudoalteromonas* genus presented, in general, lower optimal temperatures and pH values than the whole-cell biocatalysts reported in the present study. However, these have mostly originated from bacteria isolated from cold environments. For instance, a GDSL esterase from *Pseudoalteromonas* sp. 643A, isolated from the stomach of krill recovered in Antarctica, presented maximum activity at 35 °C, with a reduced residual activity at 60 °C of 20%, while showing a pH optimum at pH 8 and no activity at pH 10 [128]. A similar activity/temperature profile was reported for a lipase from *Pseudoalteromonas* sp. NJ 70, isolated from Antarctic sea ice [129]. Esterase EstO, from a psychrotolerant strain of *P. arctica*, showed its highest activity at pH 7.5, and presented a temperature optimum at 25 °C, retaining nearly 50% of its activity at 0 °C, but only 20% at 40 °C [130]. Lipases Lip1 from *P. haloplanktis* [131] and Lip1233 from *P. lipolytica* [132] showed higher optimal temperatures of 40 °C and 45 °C, respectively, but still lower than those observed in isolates 701, 705, and 729. However, Lip1 showed highest activity at pH 9, higher than the remaining lipases from *Pseudoalteromonas*, although no results for pH higher than pH 9.5 were reported in the work [131].

### 3.3. Genomic Analysis

Whole-genome sequencing of isolates 14 and 790 was carried out, to allow for the identification of putative genes encoding for cell-associated lipolytic enzymes. Members of the genus *Serratia* have emerged as promising candidates for the production of hydrolytic enzymes for industrial application due to their activity and stability. In our study, the *S. quinivorans* strain allowed the highest biomass concentration, the highest tributyrin activity, and ca. 80% of the best activity in trioctanoin (Table 2). On the other hand, strain *P. celer* was preferred over *Pseudoalteromonas* strains due to the difficulty in growing the latter strains in culture medium more appropriate to industrial applications than marine medium.

De novo assembly, to the contig level, was performed with CLC Genomics workbench (version 12.0.3), followed by feature annotation, performed on the Bakta Web platform [64] (Table 3). Annotated assemblies are archived in the ENA database, accession number GCA_982482815 for isolate 14, and accession number GCA_982482235 for isolate 790.

Coding sequences annotated as encoding for lipases, esterases, α/β hydrolases, and GDSL lipases/esterases, or variations of these annotations, were selected for further characterization of their hypothetical products in terms of function, subcellular location, and similarity to other published sequences (Supplementary Table S1). BLASTP searches of sequences of the predicted product revealed high similarity (>96% sequence identity) to sequences deposited on the “non-redundant protein sequences” database, belonging to strains of the genera *Psychrobacter*, for sequences from isolate 14, and *Serratia*, for sequences from isolate 790, indicating that these products may be conserved at the species level, and potentially at the genus level.

In addition to their annotation with Bakta, hypothetical gene products were classified with the HMMER tool, available in the ESTHER database of the α/β hydrolase superfamily of enzymes. This tool allows for the classification of an enzyme’s subfamily, as defined in the database, according to its homology to hidden Markov model profiles generated for each subfamily [65]. The correspondence between selected α/β hydrolase subfamilies in the ESTHER database and the bacterial lipolytic enzyme classification system by Arpigny and Jaeger [72], since expanded by Kovacic et al. [9], has been described previously [66], and was included in the analysis. It should be noted that bacterial lipolytic enzyme families II and VIII do not belong to the α/β hydrolase superfamily of enzymes [9], and are therefore not included in the ESTHER database [66]. Additionally, despite including enzymes with phospholipase activity, the bacterial lipolytic enzyme classification system does not explicitly include phospholipases [9]. Several enzymes annotated as phospholipases could not be classified with the HMMER tool, probably due to not being α/β hydrolases, and thus not being included in the scope of the database. For instance, both isolates presented putative genes predicted to encode for outer membrane phospholipase A1 (Pclip-8 and Sqlip-15, Table 4), a class of dimeric phospholipases in Gram-negative bacteria [133], that has been shown to be active (in *E. coli*) towards mono- and di-acylglycerols, but not TAG [134]. The HMMER tool could not classify either putative enzyme. In such cases, an additional search of the sequences with the InterProScan tool [67,68] was performed.

Only a fraction of the features analysed with the HMMER/InterProScan tools could be classified as lipolytic enzymes (Supplementary Table S1), as expected, given the variety of functions performed by the members of the α/β hydrolase superfamily of enzymes [135]. The subcellular location of the lipolytic enzymes remained a significant question, given the lack of free lipase activity observed during the screening assays. Two tools were applied to help in answering that question: DeepLocPro, a tool relying on a machine learning algorithm to predict the subcellular location of prokaryotic proteins [69]; and SignalP, to predict signal peptides [70]. Both isolates presented putative genes encoding for lipolytic enzymes with predicted extracellular or outer membrane locations (Table 4).

The genome of isolate 14 presented three putative genes predicted to encode for lipolytic enzymes with extracellular locations. Pclip-6 was classified by the HMMER tool as a member of lipolytic enzyme family I.1, a subfamily of the “true lipases” [9]. A common, but not ubiquitous [136], characteristic of enzymes belonging to this subfamily, and subfamily I.2, is the requirement of specific periplasmatic chaperones, or Lif proteins, for the production of the correctly folded, active lipases [9,137,138]. The coding sequences for these chaperones are often found near their cognate lipase genes, with both genes potentially encoded on an operon [137]. The genome of isolate 14 presented a putative coding sequence for a “lipase secretion chaperone” 60 bp upstream of the hypothetical coding sequence for Pclip-6. InterProScan analysis of the coding sequence product similarly predicted its function as a lipase secretion chaperone. The close proximity between the two sequences, and the classification of Pclip-6 as family I.1 lipase, indicate that they are likely a lipase-periplasmatic chaperone pair. Although Pclip-6 was predicted by DeepLocPro to be an extracellular enzyme, it should be noted that non-covalent associations of extracellular lipases with lipopolysaccharides, for instance in *P. aeruginosa* [139,140], have also been observed previously, and so enzymes directly anchored to cell membranes may have not been the sole biocatalysts responsible for the cell-bound activity detected in this work.

A second hypothetical enzyme, Pclip-28, was classified as belonging to family IV of lipolytic enzymes. Kovacic et al. described this family of bacterial enzymes as only containing esterases, a significant distinction from their homologous mammalian hormone sensitive lipases [9,72,141]. Nevertheless, several studies have reported the hydrolysis of *p-*nitrophenyl esters of medium- and long-chained fatty acids by members of this family [142,143,144], although these substrates have been found to sometimes be poor analogues for acylglycerols composed by the same fatty acids [145,146]. Therefore, expression of Pclip-28, and its characterization in terms of substrate selectivity towards acylglycerols containing long-chain fatty acids, would be required to confirm if it has a role in the observed cell-associated lipase activity of isolate 14, which is also true for all other predicted enzymes presented in this work. A third lipolytic enzyme, Pclip-3, classified as belonging to the lipolytic enzyme family X.2 [9,147], was predicted by DeepLocPro to be an extracellular enzyme, although at only a slightly higher probability than it being an outer membrane protein. However, the prediction of a lipoprotein signal peptide by SignalP may indicate that Pclip-3 is an outer membrane-anchored enzyme, presenting its hydrolytic domain in the periplasm, given this is the most common location for lipoproteins in Gram-negative bacteria, although translocation of lipoproteins to the cell surface is also known to occur [148,149].

The genome of isolate 790 presented a single putative extracellular lipolytic enzyme, Sqlip-4, classified as belonging to lipase subfamily I.3. This subfamily of “true lipases” is distinguished from subfamilies I.1 and I.2 not only by the low sequence similarities with enzymes from those subfamilies, but also by relying on the type I secretion pathway, instead of the type II secretion pathway used by lipases from subfamilies I.1 and I.2 [23,150]. This is exemplified by the lack of an N-terminal signal peptide [150] in Sqlip-4, such as the one predicted by SignalP in Pclip-6. Several enzymes belonging to this subfamily have been found to present polyurethanase activity [127,151,152], making these enzymes potentially valuable in plastic waste management. Targeted hydrolysis of urethane and ester bonds in polyurethane by esterases, cutinases, and novel urethanases are required, and whole-cell biocatalysts could represent a cheaper alternative to the use of purified enzymes. Isolate 790 presented an additional putative outer membrane lipolytic enzyme, Sqlip-20, annotated by Bakta as “Lipase1”, corresponding to entry K12686 (named “outer membrane lipase/esterase”) in the KEGG orthology database, associated with enzymes belonging to the GDSL hydrolase (or SGNH hydrolase) family of lipolytic enzymes (family II) [9,103,153]. Accordingly, the HMMER tool did not classify Sqlip-20. InterProScan classified Sqlip-20 as belonging to the family of the EstA lipases with autotransporters, identifying an SGNH hydrolase domain and an autotransporter domain, responsible for mediating the translocation of the passenger domain through the outer membrane of the cell [154]. GDSL hydrolases present the peculiarity that the passenger domain remains attached to the autotransporter domain after translocation (unlike what is often observed in other autotransporter-containing proteins), thus anchoring the enzyme to the cell surface [155]. GDSL hydrolases in general [156,157], and GDSL hydrolases with autotransporter domains in particular [155], show large variability in substrate selectivity, allowing for the possible classification of these enzymes as esterases, lipases, and/or phospholipases [157].

## 4. Conclusions

Marine environments have proven to be valuable sources of bioproducts and biocatalysts. In this work, 94 isolates, mostly originating from marine environments, were screened for the production of lipases. A total of 25% of the screened isolates presented cell-bound lipolytic activity, as determined by the hydrolysis of the lipase substrate trioctanoin. Six of these isolates, originating from temporary rock pools, presented the highest lipolytic activity. These isolates were further characterized as whole-cell biocatalysts. In concordance with varying temperatures and media composition imposed by their natural environment, these isolates presented activity at broad ranges of pH and temperature. These tolerances can be invaluable in industrial applications. However, further characterization of these biocatalysts, e.g., the evaluation of their affinity towards industrially relevant substrates, and of their stability under reactor conditions and in the organic solvent systems required for (trans)esterification reactions, is still required for the assessment of their applicability to industrial processes.

The analysis of the genomes of two of the most-active isolates revealed the presence of putative sequences encoding for conventional, extracellular, true lipases, but also for hypothetical lipolytic enzymes covalently bonded to membrane anchors, which may be responsible for the observed cell-bound activity. It must be noted that the expression of none of these predicted lipolytic enzymes was experimentally validated, with the presence of extracellular lipase activity on liquid culture biomass being the only indicator of the possibility of the expression of one (or more) of these proteins. Additional work, focusing on the isolation and identification of membrane-bound enzymes in these isolates, and/or the expression of the putative lipase/esterase genes in exogenous hosts, should be performed in the future to validate the expression, subcellular location, and activity of the putative lipolytic enzymes proposed in this study. Still, the objective of this study—identification of novel lipolytic biocatalysts—was achieved, with the selection of six lipolytic whole-cell biocatalysts, with wide temperature and pH activity ranges. In fact, the whole-cell may protect the enzymes from harsh environmental/reaction conditions, which could result in wider range of applications. Additionally, the complementary genomic analysis revealed the sequences needed for recombinant lipase production, enabling a bridge between purified enzyme catalysis strategies and whole-cell biocatalysts.

## Figures and Tables

**Table 1:** Sources of isolates screened for production of lipases.

Isolate Number	Sampling Site				References
	Location	GPS Coordinates (N, E)	Sampling Date (d-m-y)	Type of Site	
2; 14; 19	Guincho	38.7185430, −9.4805880	8-09-2015	Rock pool	(this study)
700–706; 708–713; 716; 720–723; 725–731; 733; 736; 739–745; 747; 750–755; 758–760; 769–771; 773–776; 779; 781; 785; 790–793; 795–798; 805; 807–809	Guincho	38.7185430, −9.4805880	22-02-2022	Rock pool	(this study)
878; 882; 883; 898–901	Salinas do Samouco	38.7352542, −8.9981561	14-09-2022	Saltern crystallization pool	[51]
903	Salinas do Samouco	38.7356635, −8.9976888	14-09-2022	Saltern evaporation pool	[51]
32	Praia dos Aveiros	37.082674, −8.231876	14-09-2015	Salt precipitate from dried rock pool	(this study)
232; 233	Ponda do Castelo	37.8908333, −25.8258333	1 to 3-12-2015	Intertidal pool	[53]
282	Lagoa das Sete Cidades (Lagoa Verde)	37.8549027, −25.78583333	1 to 3-12-2015	Freshwater lake	[53]
323	Furnas	37.77323611, −25.30359167	1 to 3-12-2015	Volcanic water	[53]
346; 347; 348; 350	Caldeirão, Furnas	37.77276944, −25.304175	1 to 3-12-2015	Volcanic water	[53]
373; 375	Ribeira Quente	37.73, −25.30861111	1 to 3-12-2015	Sea water	[53]
414	Faial da Terra	37.74, −25.19527778	1 to 3-12-2015	Sea water	[53]
432	Fumarolas da Lagoa das Furnas	37.76865, −25.33173611	1 to 3-12-2015	Volcanic mud and water	[53]
457	Lagoa das Sete Cidades (Lagoa Verde)	37.85451111, −25.78588889	1 to 3-12-2015	Freshwater spring	[53]
562	Piscinas Naturais dos Mosteiros	37.88784444, −25.8225	1 to 3-12-2015	Intertidal pool	[53]
603	Poça da Dona Beija	37.76928056, −25.31935556	1 to 3-12-2015	Hot water spring	[53]
255	Lagoa das Sete Cidades (Lagoa Azul)	37.865425, −25.79267778	1 to 3-12-2015	Freshwater lake	[53]

**Table 2:** Identification of six isolates, selected for presenting high activity with tributyrin/trioctanoin. Correspondent characterization of the whole-cell biocatalysts following liquid cultivation in the mentioned media. The cell growth rate is represented by µ. Culture volumetric activity indicates the number of lipase units produced per litre of culture, as determined by the specific activity of the whole-cell biocatalysts at pH 8, 30 °C. All assays were performed in duplicate.

Isolate		Culture Parameters			Culture Volumetric Activity, U L^−1^		
Number	Identification (16S rDNA)	Culture Media	μ, h^−1^	Biomass Concentration, g_dry weight_ L^−1^	Tributyrin	Trioctanoin	*p*NPB
14	*Psychrobacter celer*	MB	0.648	0.965	23.34	6.07	20.82
701	*Pseudoalteromonas espejiana*	MB	0.667	0.73	35.98	16.96	98.05
705	*Pseudoalteromonas undina*	MB	0.654	0.98	41.99	14.18	106.75
725	*Psychrobacter* sp.	MB	0.527	0.814	13.28	7.94	16.31
729	*Pseudoalteromonas undina*	MB	0.574	1.172	39.37	16.45	100.84
790	*Serratia quinivorans*	SPGY	0.459	2.627	78.22	12.95	76.61

**Table 3:** Summary of the genome assemblies and annotations for isolates 14 and 790.

		Isolate	
		*P. celer* (Isolate 14)	*S. quinivorans* (Isolate 790)
**Assembly**	GC content (%)	47.35%	55%
	Assembly length (bp)	3,027,584	5,263,968
	Number of Contigs	47	64
	Contig L50	6	10
	Contig N50 (bp)	156,610	165,162
**Feature annotation**	tRNAs	43	73
	rRNAs	3	6
	coding DNA sequences	2539	4807

**Table 4:** Putative lipolytic enzymes from isolates 14 and 790, with predicted extracellular or outer membrane locations. Lipolytic enzyme family was predicted with the HMMER tool from the ESTHER database. Accession numbers (NCBI Protein database) for proteins with the highest relative identity to the sequence of the corresponding putative lipase are also presented.

Isolate	Putative Enzyme	Locus Tag	Lipolytic Enzyme Family (HMMER)	InterProScan Protein Family	DeepLocPro (%)			SignalP	BLASTP	
					Other Locations	Extracellular	Outer Membrane		Accession Number	Identity (%)
14	Pclip-3	OKPAKJ_01445	X.2		10.8	48.4	40.8	Lipoprotein signal peptide (Sec/SPII)	MDN5733541.1	99.46
	Pclip-6	OKPAKJ_02058	I.1		0.3	99.2	0.6	Signal Peptide (Sec/SPI)	WP_289056820.1	100.00
	Pclip-8	OKPAKJ_02434		Phospholipase A1	3.0	0.1	96.9		WP_348548957.1	99.80
	Pclip-28	OKPAKJ_02523	IV		31.6	63.0	5.4	Signal Peptide (Sec/SPI)	WP_256713787.1	98.22
790	Sqlip-4	PGGIOI_00205	I.3		0.2	99.6	0.2		WP_135344359.1	100.00
	Sqlip-15	PGGIOI_03132		Phospholipase A1	0.4	0.0	99.6	Signal Peptide (Sec/SPI)	WP_012004663.1	100.00
	Sqlip-20	PGGIOI_04284		Lipase, autotransporter EstA; GDSL lipase/esterase	3.9	6.3	89.8	Signal Peptide (Sec/SPI)	WP_261132755.1	99.24

## Data Availability

The original contributions presented in this study are included in the article/Supplementary Materials. Annotated genomes presented in this study are openly available in the European Nucleotide Archive with accession numbers GCA_982482815 and GCA_982482235. Further inquiries can be directed to the corresponding author.

## Supplementary Materials

The following supporting information can be downloaded at: https://www.mdpi.com/article/10.3390/microorganisms14061355/s1; Table S1: Selection of annotated features, classified as lipolytic enzymes, from the genomes of isolates 14 and 790, including product function and cell localization predictions. The ESTHER HMMER predictions for family classification correspond to the predictions presenting the highest score. BLASTP results are for the sequence present on the non-redundant protein sequences database presenting the highest identity with the query sequence. Annotated assemblies are archived in the ENA database, accession numbers GCA_982482815 for isolate 14, and accession number GCA_982482235 for isolate 790.

## Abbreviations

The following abbreviations are used in this manuscript:

bp	Base pair
CAPS	3-(Cyclohexylamino)-1-propanesulfonic acid
CHES	2-(Cyclohexylamino)ethanesulfonic acid
DMSO	Dimethyl sulfoxide
EPPS	4-(2-Hydroxyethyl)-1-piperazinepropanesulfonic acid
FAME	Fatty acid methyl ester
GC–FID	Gas chromatograph–flame ionization detector
MA	Marine agar
MB	Marine broth
MES	2-Morpholinoethanesulfonic acid monohydrate
PCA	Principal component analysis
PHIBLA	pH indicator-based lipase assay
PIPES	1,4-Piperazinediethanesulfonic acid
*p*NPB	*p*-Nitrophenyl butyrate
Tris	Tris(hydroxymethyl)aminomethane
TSA	Trypticase soya broth
TSB	Trypticase soya agar
UV	Ultraviolet
