# Supplementary material for: Screening of Marine Bacteria for Lipase Activity and Application as Whole-Cell Biocatalysts
Source: Microorganisms. 2026 Jun 17;14(6):1355. doi: 10.3390/microorganisms14061355 (PMC13304423; doi:10.3390/microorganisms14061355)
Supplement: Supplementary file 1 [file microorganisms-14-01355-s001.zip › microorganisms-4343934-supplementary.pdf]

Table S1 – Selection of annotated features, classified as lipolytic enzymes, from the genomes of isolates 14 and 790, including product function and cell localization predictions. The ESTHER HMMER predictions for family classification correspond to the predictions presenting the highest score. BLASTp results are for the sequence present on the non-redundant protein sequences database presenting the highest identity with the query sequence. Annotated assemblies are archived in the ENA database, accession numbers GCA_982482815 for isolate 14, and accession number GCA_982482235 for isolate 790.

Isolate	Locus tag	Putative enzyme	Annotation (Bakta)	**ESTHER HMMER**				InterProScan protein family	**DeepLocPro cellular location prediction (%)**						SignalP	**BLASTp (non-redundant protein sequences database)**			
				Family classification	Lipolytic enzyme family	Score	E-value		Cell wall & surface	Extracellular	Cytoplasmic	Cytoplasmic Membrane	Outer Membrane	Periplasmic		Accession number	Description	Identity (%)	Species
Psychrobacter celer (Isolate 14)	OKPAKJ_00393	Pclip-1	lipase	Hormone-sensitive_lipase_like_1	Family IV	236.8	8E-70		0.0	6.8	76.5	13.9	1.9	0.8		WP_443551519.1	alpha/beta hydrolase	0.9831	*Psychrobacter celer*
	OKPAKJ_00791	Pclip-2	carboxylesterase	Lysophospholipase_carboxylesterase	Family VI	143.3	1.1E-41		0.0	0.0	99.4	0.0	0.1	0.4		WP_443550961.1	alpha/beta hydrolase	0.9955	*Psychrobacter celer*
	OKPAKJ_01445	Pclip-3	Secretory lipase	Fungal-Bact_LIP	Family X.2	8.1	5E-11		1.5	48.4	1.5	6.0	40.8	1.7	Lipoprotein signal peptide (Sec/SPII)	MDN5733541.1	alpha/beta fold hydrolase	0.9946	*Psychrobacter* sp.
	OKPAKJ_02058	Pclip-6	AB hydrolase-1 domain-containing protein	Bacterial_lip_FamI.1	Family I-1	536.9	3.7E-160		0.0	99.2	0.1	0.1	0.6	0.1	Signal Peptide (Sec/SPI)	WP_289056820.1	esterase/lipase family protein	1	*Psychrobacter*
	OKPAKJ_02434	Pclip-8	Phospholipase A1	Polyesterase-lipase-cutinase	Family III	-160.6	0.44	Phospholipase A1	0.0	0.1	0.3	2.2	96.9	0.5		WP_348548957.1	phospholipase A	0.998	*Psychrobacter* sp. KFRI-CH2-11
	OKPAKJ_00935	Pclip-18	G-D-S-L lipolytic protein					Thioesterase 1/Protease 1/Lysophospholipase L1	0.0	0.1	28.2	67.8	3.2	0.7		WP_348549332.1	SGNH/GDSL hydrolase family protein	0.9926	*Psychrobacter* sp. KFRI-CH2-11
	OKPAKJ_02523	Pclip-28	Alpha/beta hydrolase	Hormone-sensitive_lipase_like_1	Family IV	28.2	6E-10		1.0	63.0	1.7	7.0	5.4	22.0	Signal Peptide (Sec/SPI)	WP_256713787.1	alpha/beta hydrolase	0.9822	*Psychrobacter* sp. Rd 27.2
	OKPAKJ_00502	Pclip-31	Alpha/beta hydrolase	Carboxymethylbutenolide_lactonase	Family V.2	-28.6	2.2E-07		0.0	0.2	31.2	65.8	2.2	0.6		WP_443575896.1	alpha/beta hydrolase family protein	0.9868	*Psychrobacter celer*
	OKPAKJ_01506	Pclip-35	Alpha/beta hydrolase	Carboxymethylbutenolide_lactonase	Family V.2	268	3.1E-79		0.6	3.2	79.2	12.8	1.8	2.4		WP_075101060.1	alpha/beta fold hydrolase	1	*Psychrobacter*
	OKPAKJ_01543	Pclip-36	Alpha/beta hydrolase	Carboxymethylbutenolide_lactonase	Family V.2	-23	1E-07		0.0	0.1	8.8	89.3	1.6	0.2		WP_100749229.1	alpha/beta fold hydrolase	0.9967	*Psychrobacter sp.* L7

Serratia quinivorans (Isolate 790)	PGGIOI_00205	Sqlip-4	Triacylglycerol lipase	Bacterial_lip_FamI.3	Family I.3	1287.5	0		0.2	99.6	0.0	0.0	0.2	0.0		WP_135344359.1	Polyurethane esterase	1	*Serratia*
	PGGIOI_00787	Sqlip-6	Lipase 2	Hormone-sensitive_lipase_like_1	Family IV	227.8	4E-67		0.0	0.2	98.9	0.1	0.3	0.4		WP_012145575.1	alpha/beta hydrolase	1	*Serratia*
	PGGIOI_01147	Sqlip-7	Esterase/lipase-like protein	Hormone-sensitive_lipase_like_1	Family IV	-22.5	4.5E-06		0.0	1.1	96.4	0.0	0.1	2.4		WP_115184348.1	alpha/beta hydrolase	0.9962	*Serratia*
	PGGIOI_02299	Sqlip-12	Lipase 2	Hormone-sensitive_lipase_like_1	Family IV	256.6	8.7E-76		0.0	1.7	96.1	0.0	0.1	2.1		WP_017892938.1	alpha/beta hydrolase	0.9737	*Serratia* sp. S4
	PGGIOI_03132	Sqlip-15	phospholipase A					Phospholipase a1	0.0	0.0	0.0	0.2	99.6	0.3	Signal Peptide (Sec/SPI)	WP_012004663.1	phospholipase A	1	*Serratia*
	PGGIOI_04284	Sqlip-20	Lipase 1	Duf_3530		-72.8	0.024	Lipase, autotransporter EstA; GDSL lipase/esterase	0.1	6.3	0.0	0.1	89.8	3.7	Signal Peptide (Sec/SPI)	WP_261132755.1	autotransporter outer membrane beta-barrel domain-containing protein	0.9924	*Serratia quinivorans*
	PGGIOI_00053	Sqlip-24	Acetyl esterase	Hormone-sensitive_lipase_like_1	Family IV	11.8	1.1E-08		0.0	5.9	83.5	0.3	0.3	10.0		SUI91250.1	Acetyl esterase	1	*Serratia quinivorans*
	PGGIOI_00107	Sqlip-25	Tropinesterase	Carboxymethylbutenolide_lactonase	Family V.2	94.9	4.2E-27		0.0	2.7	91.6	1.5	0.3	4.0		WP_012145038.1	alpha/beta fold hydrolase	1	*Serratia*
	PGGIOI_00949	Sqlip-32	esterase	Lysophospholipase_carboxylesterase	Family VI	68.4	4E-19		0.0	0.0	99.6	0.2	0.1	0.0		WP_218217458.1	esterase	1	*Serratia*
	PGGIOI_01386	Sqlip-38	esterase	Carboxymethylbutenolide_lactonase	Family V.2	121.9	3.1E-35		0.0	0.1	99.7	0.0	0.1	0.1		WP_112364355.1	esterase	1	*Serratia*
	PGGIOI_01409	Sqlip-40	bifunctional 2',3'-cyclic-nucleotide 2'-phosphodiesterase/3'-nucleotidase	Bacterial_lip_FamI.3	Family I.3	-257.5	0.065		0.0	1.4	0.0	0.0	0.2	98.3	Signal Peptide (Sec/SPI)	WP_259191786.1	bifunctional 2',3'-cyclic-nucleotide 2'-phosphodiesterase/3'-nucleotidase	0.9969	*Serratia* sp. BIGb0163
	PGGIOI_02660	Sqlip-45	enterochelin esterase	Lysophospholipase_carboxylesterase	Family VI	28.5	1E-08		0.0	13.0	82.7	2.6	0.1	1.6		WP_037410468.1	enterochelin esterase	0.9934	*Serratia*
	PGGIOI_03002	Sqlip-50	alpha/beta hydrolase	Lysophospholipase_carboxylesterase	Family VI	-12.7	0.001		0.0	1.5	2.4	0.1	0.6	95.4	Signal Peptide (Sec/SPI)	WP_112364180.1	alpha/beta hydrolase	0.9932	*Serratia quinivorans*
	PGGIOI_04327	Sqlip-64	Alpha/beta hydrolase family	Lysophospholipase_carboxylesterase	Family VI	-28.1	0.024		0.2	23.6	16.3	11.0	4.6	44.3	Signal Peptide (Sec/SPI)	WP_261132732.1	alpha/beta hydrolase	1	*Serratia*
	PGGIOI_04070	Sqlip-71	2-hydroxy-6-oxo-6-phenylhexa-2,4-dienoate hydrolase	Carboxymethylbutenolide_lactonase	Family V.2	132.8	1.6E-38		0.0	1.3	92.7	0.3	0.1	5.6		WP_261099520.1	alpha/beta fold hydrolase	0.9963	*Serratia quinivorans*

	PGGIOI_04392	Sqlip-72	Alpha/beta hydrolase fold-3 domain protein	Hormone-sensitive_lipase_like_1	Family IV	140.2	9.8E-41		0.4	2.4	43.7	46.3	4.5	2.7		WP_261100523.1	alpha/beta hydrolase	1	*Serratia quinivorans*
	PGGIOI_03507	Sqlip-74	Ketosteroid isomerase-related protein	Carboxymethyl-butenolide_lactonase	Family V.2	-22	8.8E-08		0.0	1.1	96.7	0.3	0.2	1.7		WP_476705650.1	alpha/beta hydrolase	0.9962	*Serratia* sp. AR11
